# A Five-Minute UHPLC–MS/MS Method Facilitates Anthocyanin Biofortification in Maize (*Zea mays* L.)

**DOI:** 10.3390/molecules31213920

**Published:** 2026-10-30

**Authors:** Franz J. O’Hanna, Shaji K. Chacko, Samuel D. Jesse, Alexander I. Hernandez, Wenwei Xu, Timothy Paape, Michael P. Dzakovich

**Affiliations:** 1 USDA-ARS Children’s Nutrition Research Center, Department of Pediatrics, Baylor College of Medicine, 1100 Bates Ave., Houston, TX 77030, USAschacko@bcm.edu (S.K.C.); samuel.jesse@bcm.edu (S.D.J.);; 2 Department of Soil and Crop Sciences, Texas A&M University, 370 Olsen Blvd., College Station, TX 77843, USAtimothy.paape@usda.gov (T.P.); 3 USDA-ARS Responsive Agriculture Food Systems Research Unit, 498 Olsen Blvd., College Station, TX 77843, USA

**Keywords:** high-throughput phenotyping, plant breeding, anthocyanins, phenomics, pigmented corn, multiple-reaction monitoring, chromatography, cyanidin, pelargonidin, peonidin

## Abstract

Anthocyanin-rich maize offers opportunities for nutritional biofortification and natural food colorants, but conventional chromatographic methods limit the throughput needed for large-scale breeding programs. We developed a 5-min ultra-high-performance liquid chromatography–tandem mass spectrometry (UHPLC–MS/MS) method for quantifying maize anthocyanins and compared its performance with a 25-min method. Anthocyanins from five commercial products and one breeding line were analyzed using optimized chromatographic gradients and multiple-reaction monitoring. Anthocyanin concentration and composition varied substantially among varieties, with differing contributions from cyanidin-, peonidin-, and pelargonidin-derived compounds. The 5-min method reduced analytical runtime fivefold while preserving the relative ranking of varieties by total anthocyanin concentration, although absolute concentrations differed slightly between methods for most varieties. Random-effects modeling indicated that the methodology used to phenotype maize represented less than 1% of the total variation, further reinforcing that our rapid method is suitable for profiling breeding populations. This rapid UHPLC–MS/MS method provides a high-throughput analytical platform for quantifying predominant maize anthocyanins, facilitating phenotyping and selection for anthocyanin biofortification in maize breeding programs.

## 1. Introduction

Anthocyanins, a class of flavonoids, are glycosylated anthocyanidins responsible for the red–orange to blue–purple–black coloration of some flowers, fruits, and vegetables and serve a variety of functions in plants [1,2,3]. As antioxidants, anthocyanins scavenge free radicals, reducing oxidative stress through various mechanisms [4]. The antioxidant properties of anthocyanins are known to help protect plants against abiotic and biotic stress, improve the quality attributes of livestock products, and ameliorate a variety of chronic non-communicable diseases in humans [5,6,7,8,9,10,11]. The potential utility of anthocyanins necessitates a cost-effective means of producing and extracting these molecules, including crop breeding and metabolic engineering for higher anthocyanin content in both edible and inedible plant tissues [12,13,14]. Maize (*Zea mays* L.) varieties are capable of a producing wide range of anthocyanin species and concentrations in the pericarp, aleurone, and endosperm of kernels, with reported values ranging from 0 to 1600 mg/100 g DW (dry weight) of total anthocyanins [15,16,17,18,19,20,21]. While intake recommendations have not been made in the US, a 50 mg d^−1^ serving of anthocyanins has been encouraged in China [22], and maize is regularly consumed both as a staple crop and livestock feed in various regions of the world [23,24]. In many of these regions, pigmented landraces of maize have been used for traditional food, beverages, and dyes [25,26]. Studies have reported these pigmented landraces as high-anthocyanin varieties that could be stably extracted and used as natural food dyes with extended shelf lives [21,27]. However, the majority of commercially grown maize varieties are low in anthocyanins, and high-anthocyanin varieties require breeding to be economically competitive with elite varieties on important agronomic traits, such as yield or tolerance to extreme abiotic stress [28,29,30]. Therefore, high-throughput quantification methods for maize anthocyanins are desirable to facilitate large-scale breeding programs.

Anthocyanin extracts are often quantified using liquid chromatography coupled to a detector of choice, such as a photodiode array (PDA) or tandem mass spectrometry (MS/MS). Current methods generally have a total runtime of 30–60 min, which is cost-prohibitive [31,32]. Although a few fast 5-min ultra-high-performance liquid chromatography photodiode array (UHPLC–PDA) methods have been published for anthocyanin quantification [33,34,35], to the best of our knowledge, none have been developed specifically for maize, and a simultaneously fast and more sensitive UHPLC–MS/MS method is yet to be published for any crop [36]. A fairly comprehensive set of maize anthocyanins was established in a previous study by separating anthocyanins using a long 60-min HPLC–PDA gradient and confirming the identity of each eluted peak with product ion scans and/or nuclear magnetic resonance (NMR) [32]. Since anthocyanins at low pH generally have similar absorbance around 520 nm, coelution is more problematic with a light-based detector, like a PDA, than with a mass-based detector, like MS/MS, due to spectral similarities among analytes. A fast, targeted UHPLC–MS/MS method for quantifying maize anthocyanins, enabled by previous efforts to identify all potential analytes of interest, would facilitate large-scale breeding studies for anthocyanin biofortification in maize.

To make a more cost-effective and higher-throughput maize anthocyanin quantification method, 25-min and 5-min UHPLC–MS/MS methods were developed based on the 60 min HPLC–PDA gradient [32]. Reversed-phase columns were screened for the retention and separation of maize anthocyanins. The composition and duration of the gradient were adjusted to separate anthocyanins as quickly as possible. Product ion scans were used to reaffirm analyte identity and determine elution order. Five heirloom maize varieties and one elite breeding line were used to compare the 25-min and 5-min UHPLC–MS/MS methods. Here, we detail the validation experiments used to evaluate these methods and discuss the implications for utilizing our rapid 5-min UHPLC–MS/MS method in the context of anthocyanin biofortification in maize.

## 2. Results

### 2.1. Chromatographic Gradient

To determine which column chemistry provided the best separation for anthocyanins, we explored a number of columns, including the following: Phenomenex, Kinetex C18 1.7 μm 50 × 2.1 mm and Kinetex C18 1.7 μm 100 × 2.1 mm; Waters (Milford, MA, USA), ACQUITY Premier BEH C18 1.7 μm 30 × 2.1 mm with 5 mm VanGuard FIT guard cartridge, ACQUITY Premier BEH C18 1.7 μm 50 × 2.1 mm with 5 mm VanGuard FIT guard cartridge, and ACQUITY Premier BEH C18 1.7 μm 100 × 2.1 mm with 5 mm VanGuard FIT guard cartridge. We ultimately determined that the Waters ACQUITY series provided the best analyte retention and scalability (e.g., 100 vs. 50 vs. 30 mm).

Additionally, multiple mobile phase pH targets were explored. While 2% FA is typically used for anthocyanin separation due to the low pKa of these molecules, this composition can be harsh on pumps and ion optics. We found that 0.5% FA was an acceptable compromise between chromatographic and instrument performance. Our gradients were able to separate analytes found by Chatham and colleagues, but precursor and neutral loss scans did not indicate the presence of additional molecules such as succinylated anthocyanins.

**Figure 1:**
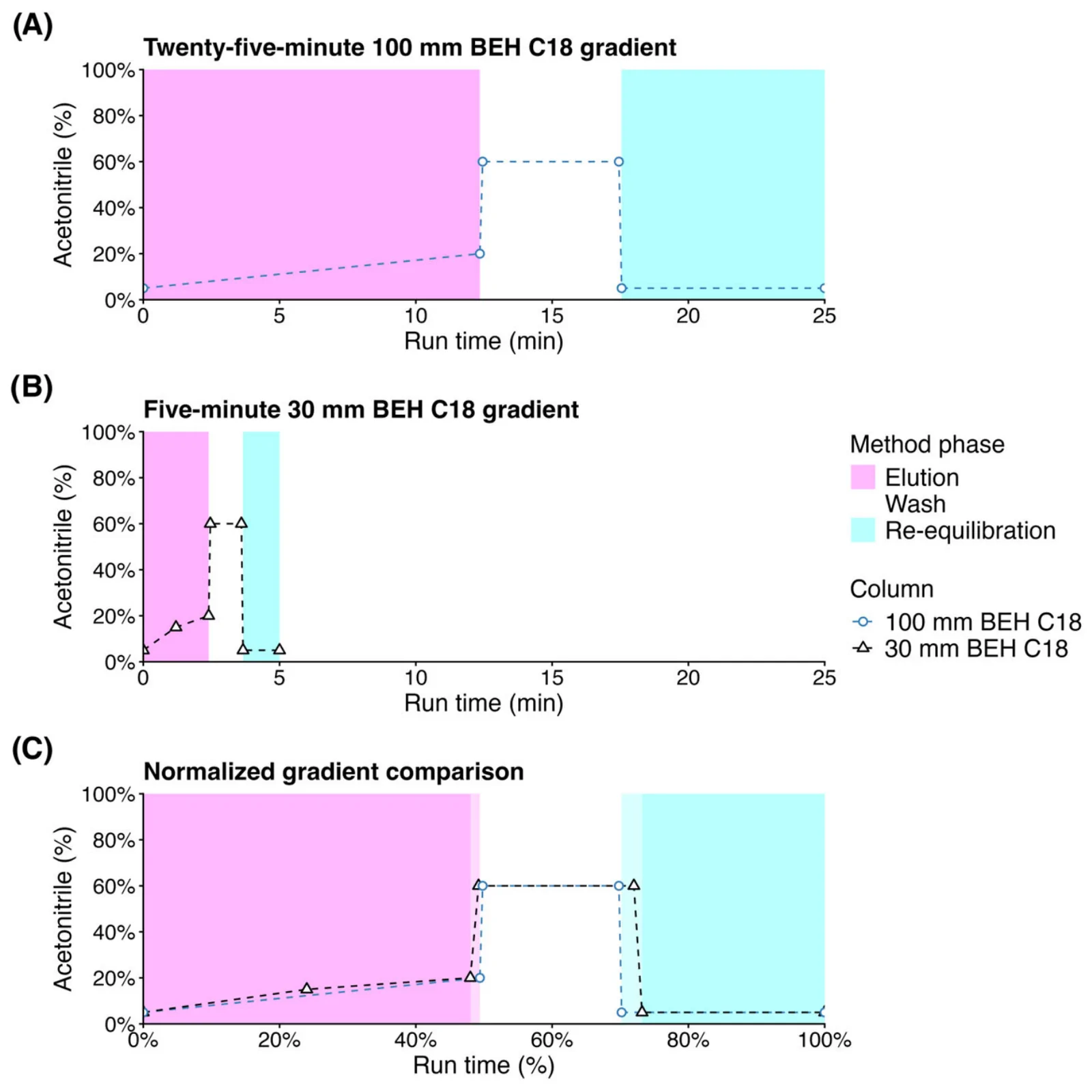
Comparison of 100 and 30 mm UPLC gradients. The percentage of mobile phase B (ACN + 0.1% FA) as a function of run time in minutes for the 100 mm and 30 mm BEH C18 gradients (**A**,**B**). Method phases are highlighted by color (elution in magenta, wash is transparent, re-equilibration in cyan). The percentage of mobile phase B for both gradients normalized by run time is visualized in (**C**). Method phases are highlighted by color (elution in magenta, wash is transparent, re-equilibration in cyan).

The gradients developed in this study are visualized in Figure 1. We displayed both the 100 mm (25 min; Figure 1A) and 30 mm (5 min; Figure 1B) methods to highlight the stark contrast in run time between these gradients. While the columns used are part of the same product line, we found that minor adjustments needed to be made to optimize chromatographic separation of maize anthocyanins. Accordingly, Figure 1C highlights the minor differences in percent mobile phase B (ACN + 0.1% FA) between the methods. This difference helped ensure sufficient separation and column cleaning for the 30 mm method. Chromatograms for each analyte run on the 30 mm (5-min) or 100 mm (25-min) method are available in Supplemental Figure S2.

### 2.2. Method Comparison

**Figure 2:**
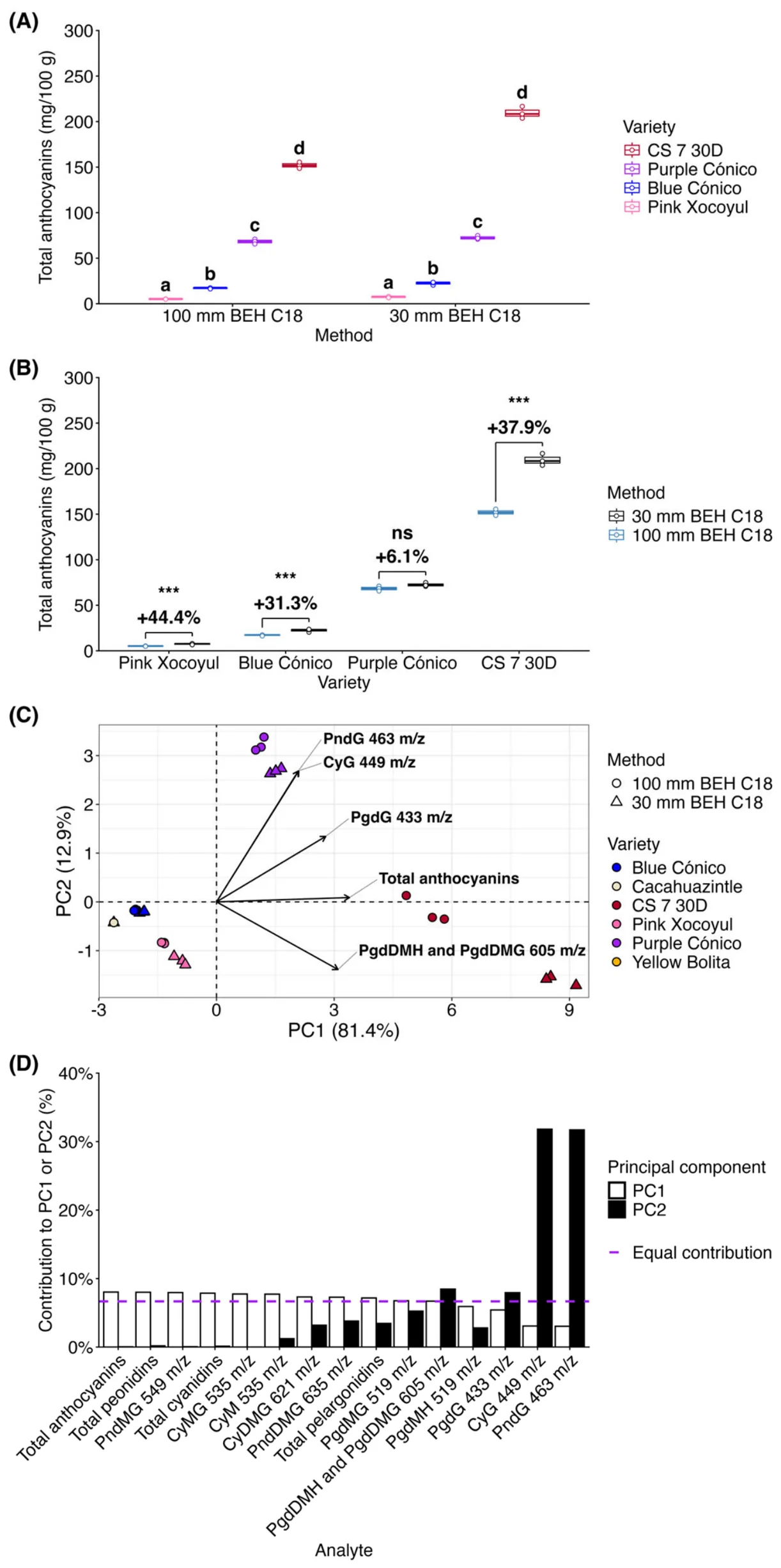
Comparison of anthocyanin profiles and concentrations in maize varieties analyzed using 30 mm and 100 mm chromatographic methods. (**A**) Principal component analysis (PCA) of quantified anthocyanins across maize varieties and chromatographic methods. Colors distinguish maize varieties, while symbols identify the analytical method (30 mm BEH C18 or 100 mm BEH C18). Arrows indicate the direction and magnitude of variable loadings on the first two principal components (PC1 and PC2), which explain 81.4% and 12.9% of the total variance, respectively. (**B**) Percentage contribution of individual anthocyanins and anthocyanin groups to PC1 (white bars) and PC2 (black bars). The dashed vertical line indicates the expected contribution of each variable under equal contribution. (**C**) Total anthocyanin concentrations (mg/100 g) across maize varieties measured using each chromatographic method. Different lowercase letters indicate statistically significant differences among varieties within the same method (*p* < 0.05). (**D**) Comparison of total anthocyanin concentrations between chromatographic methods within each maize variety. Percentages indicate the relative difference in concentrations measured using the 30 mm method compared with the 100 mm method. The dashed line represents the inflection point at which variables contributed equally to PC1 and PC2. Statistical significance is indicated by ns (not significant) and *** (*p* < 0.001). Statistical significance was determined using ANOVA followed by Tukey’s HSD (⍺ = 0.05). Three independent extractions were conducted for each maize variety profiled here.

Principal components analysis visualized anthocyanin variability as a function of variety and method and illustrated variety and method clustering (Figure 2A). Replicates of each method and variety combination clustered together. Clustering of anthocyanins by method varied across variety. Yellow Bolita, Cacahuazintle, Blue Cónico, and Pink Xocoyul were most closely clustered, while Purple Cónico and CS 7 30D (one of the hybrid breeding lines evaluated here) clustered independently from all other varieties. For each variety containing detectable anthocyanins (neither Yellow Bolita nor Cacahuazintle), replicates of each method clustered separately—particularly CS 7 30D. Loadings were added to visualize anthocyanins, or groups of anthocyanins, that contributed the most to PC1 or PC2. PC1 explained most of the variability (81.4%), and the separation of all data along PC1 is summarized by the total anthocyanins loading. The separation of CS 7 30D and Pink Xocoyul based on the method was aligned with the pelargonidin dimalonylglucoside loading. The clustering of Purple Cónico was aligned with CyG and PndG loadings, and CyG and PndG were also responsible for the majority of the contribution to PC2 (Figure 2B).

Figure 2B shows the contribution of anthocyanins, or groups of anthocyanins, to PC1 and PC2. Total anthocyanins, total peonidins, peonidin malonylglucosides, total cyanidins, cyanidin malonylglucosides, cyanidin dimalonylglucosides, peonidin dimalonylglucosides, total pelargonidins, and pelargonidin malonylglucosides all contributed more to PC1 than expected. Pelargonidin dimalonylglucosides, pelargonidin 3-glucoside, cyanidin 3-glucoside, and peonidin 3-glucoside all contributed more to PC2 than expected.

Figure 2C shows that users can arrive at the same outcome in distinguishing the order of lowest to highest anthocyanin maize varieties regardless of the method employed. Figure 2D displays the differences between varieties as a function of method. We found that the majority of the varieties profiled here were significantly different from one another, although Purple Cónico was not despite being subject to the same extraction conditions. Most of the anthocyanins in this variety are represented by the 3-glucosides that we have external calibration standards for (e.g., cyanidin-3-glucoside). Additionally, anthocyanins with isomers that would partially co-elute in the 30 mm method were not major components of its profile.

### 2.3. Range of Quantified Anthocyanins Across Varieties

**Figure 3:**
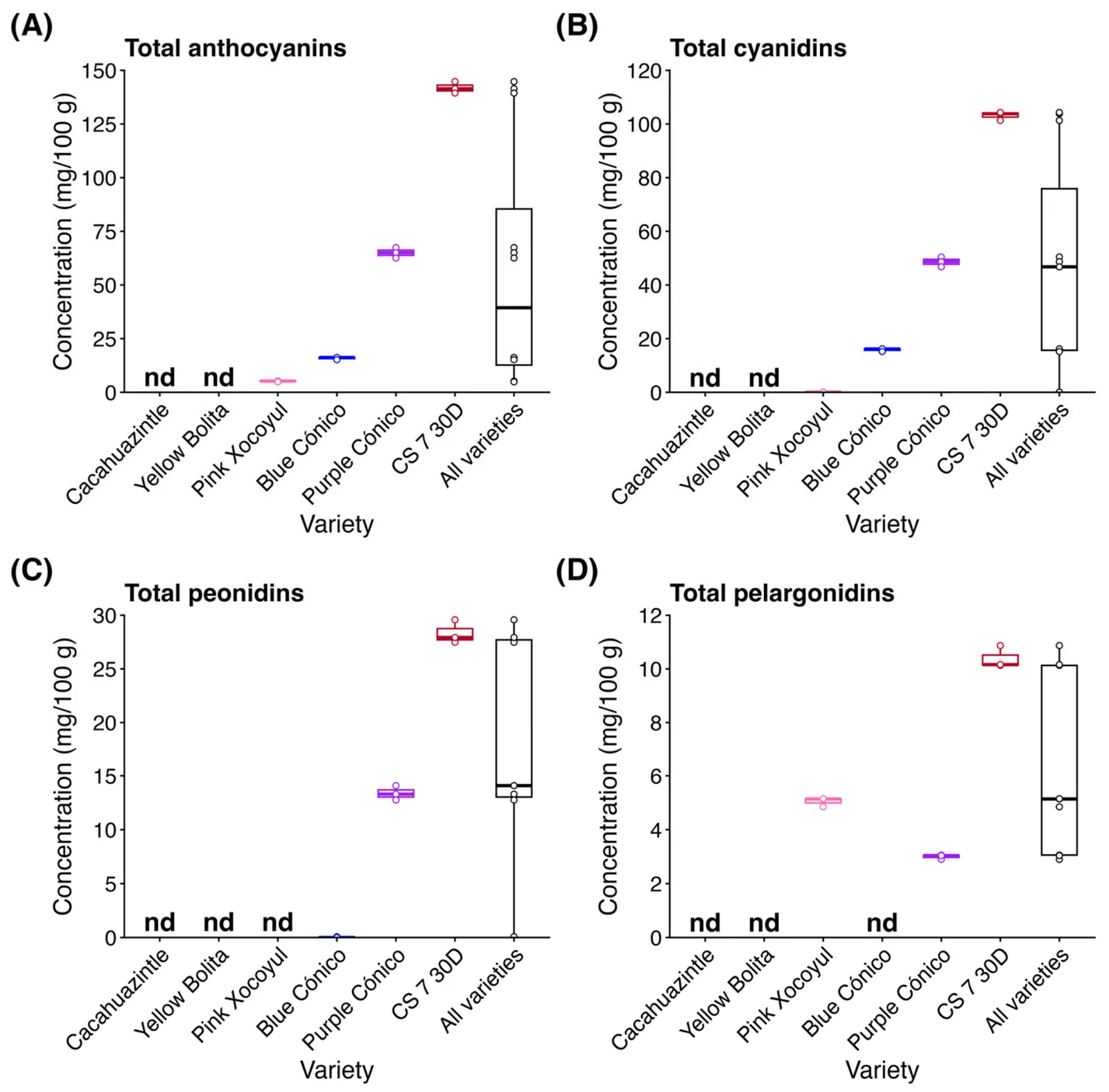
Diversity and range of quantified anthocyanin concentrations across maize varieties. Boxplots illustrate the concentrations (mg/100 g) of (**A**) total anthocyanins, (**B**) total cyanidins, (**C**) total peonidins, and (**D**) total pelargonidins in six maize varieties and across all varieties combined. Colored boxplots represent individual varieties, while black boxplots summarize the distribution across all six varieties. Data represent three independent extractions per variety analyzed. The abbreviation “nd” indicates that the corresponding anthocyanin or anthocyanin group was not detected in a given variety.

Among the tested varieties, Pink Xocoyul, Blue Cónico, Purple Cónico, and CS 7 30D had the lowest to highest total anthocyanin content, respectively (Figure 3A). Cacahuazintle and Yellow Bolita are white and yellow maize varieties, respectively, and did not contain any detected maize anthocyanins. Figure 3B–D show quantified concentrations of anthocyanins grouped by their respective anthocyanidins. Additional box-and-whisker plots for individual anthocyanins quantified using our methodology can be found in Supplemental Figure S1. In the sample material surveyed here, total anthocyanins were present at concentrations of up to 150 mg/100 g DW. On average, total cyanidins, followed by total peonidins, and, lastly, total pelargonidins represented the highest to lowest share of total anthocyanins. However, only Purple Cónico and CS 7 30D independently followed this trend. Pink Xocoyul was high in pelargonidins, low in cyanidins, and did not contain any detectable peonidins. Blue Cónico was high in cyanidins and low in peonidins, but did not contain any detectable pelargonidins. CyMG and CyDMG were the predominant cyanidins, PndMG and PndDMG were the predominant peonidins, and PgdMG and PgdDMG were the predominant pelargonidins, which supports the biological importance of anthocyanin malonylation and acylation, in general, in maize.

Notably, we were able to identify the condensed flavonol anthocyanins previously identified by Juvik and colleagues [37]. However, these analytes were only detectable when using extremely concentrated QC samples from isolated pericarp. In the material we analyzed here, these molecules were not detectable, indicating that genetic variation is highly important in controlling the profile of anthocyanins found in maize.

### 2.4. LOD/LOQ

To define the lower limits of our 5-min method, we calculated both the limit of detection (LOD) and limit of quantification (LOQ). Outcomes of these calculations are in Table 1 and indicate that our method can quantify as low as 77.71 femtomoles on the column.

### 2.5. Intraday and Interday Variability

To determine how our methodology varied within and between days, we calculated intraday and interday variability (Table 2; Supplemental Tables S3 and S4). Intra- and interday variability data generated utilizing the longer 25-min method can be found in Supplemental Table S4. While we were able to detect and optimize SRMs for many of the condensed forms of anthocyanins using concentrated pericarp material, these compounds were generally not detectable when analyzing samples created from whole grains. Intraday variability values were on average 6.4%, while interday variability values were on average 10.6%. These findings indicate that our 5-min method is reproducible and suitable for phenotyping despite some structural isomers becoming chromatographically unresolved. Users wishing to resolve these isomers can do so with the 25-min method (Supplemental Figure S2).

### 2.6. Variance Partitioning and Additional Validation

To determine how our 5-min method performs in the context of phenotyping a maize population, we profiled the maize lines described above using either the 100 mm 25-min method or 30 mm 5-min method (Supplemental Table S5). Random-effects modeling was used to determine the percent of total variation that each experimental component contributed (Table 3). Given that some of the isomers present in samples coeluted in the 5-min method, aggregate values for these masses (e.g., 519 m/z) were used for analysis. We found that genetic background explained the vast amount of total variance in the measured analytes, while the analytical method generally explained less than 1%. This finding indicates that either method would be suitable to profile a maize population, but significant time and resource savings could be capitalized upon by using the 5-min method. These findings are further supported by virtually identical phenotypic patterns of a small population (kernels collected from 11 individual plants) analyzed using the 5- and 25-min methods (Supplemental Table S6).

## 3. Discussion

### 3.1. Influence of pH and Chromatographic Conditions

Anthocyanin chromatography is strongly influenced by pH because anthocyanins undergo pH-dependent structural transitions. Strongly acidic conditions favor the colored flavylium cation, whereas increasing pH shifts the equilibrium toward other forms [38]. Acidification therefore improves sample stability, peak shape, and retention and also promotes the form that absorbs strongly near 520 nm, facilitating selective PDA/DAD (photodiode array/diode array detector) detection [36].

Detector choice ultimately dictates gradient pH and composition. PDA is well suited for rapid quantification of previously characterized anthocyanins because acidic conditions enhance visible absorbance and detection does not depend on electrospray ionization. MS/MS provides greater structural specificity, but mobile-phase composition can influence ion formation. For example, Paulsmeyer and colleagues used 5% FA for UHPLC–DAD but only 0.2% FA for LC–MS/MS identification, and trifluoroacetic acid has been reported to reduce electrospray ion yield through ion pair formation [14,36]. These differences support optimizing PDA and MS methods separately rather than assuming identical acidic conditions are ideal for both. We found that 0.5% FA provided the best compromise that limited damage to our MS platform but reduced loss of definition between peak shoulders and generally narrowed peaks. For PDA-based platforms, we recommend considering 2% FA for mobile phase A to reduce co-elution and peak broadening.

Finally, anthocyanin methods often begin under highly aqueous conditions, which can cause retention loss on some reversed-phase materials after flow interruption. This behavior is better described as pore dewetting than traditional “phase collapse” [39]. Published maize methods have used high-organic-content washes and re-equilibration between injections, and successful separations have been reported with Poroshell SB-C18 and ACQUITY BEH C18 phases under acidic conditions [14,32,40]. To reduce pore dewetting, we used a 60% ACN wash phase near the end of our gradient to keep the stationary phase hydrated, but unwanted molecules (e.g., flavonoids) that were likely retained on the column are still removed. We found that above 60% ACN, retention time shifts were noticeable in large batches of samples. If our method is used with different column chemistry, it is strongly advised to do extensive testing to ensure retention time stability.

### 3.2. Sensitivity, Analytical Throughput, and Intended Application

The five-minute UHPLC–MS/MS method demonstrated limits of detection (LOD) and quantification (LOQ) of 25.64–64.63 and 77.71–195.85 fmol/injection, respectively, corresponding to approximately 2.56–6.27 and 7.75–18.99 ng/mL using a 5 µL injection volume (Table 1). These values are substantially lower than those reported for maize anthocyanins using HPLC–DAD by Colombo and others (LOD: 40–140 ng/mL; LOQ: 150–500 ng/mL) and Nurkhasanah and colleagues (LOD: 380 ng/mL; LOQ: 1180 ng/mL for cyanidin-3-glucoside) [41,42]. Additional maize-specific analytical methods have been described by Hong et al. (2020) and Custodio-Mendoza et al. (2025), providing further context for chromatographic performance and method validation [40,43]. Although differences in detection technology, sample preparation, and reporting units limit direct comparisons, these findings demonstrate that the five-minute method provides favorable analytical sensitivity while substantially increasing sample throughput.

A principal advantage of the present method is substantially greater analytical throughput than many established maize anthocyanin methods. Previous studies commonly used gradients of approximately 30 min or longer, including 30-min HPLC–PDA separations for maize germplasm and breeding populations and a 58-min LC–MS/MS separation for structural identification [14,18,32,40]. Shorter UHPLC methods can therefore reduce solvent use and greatly increase the number of samples analyzed per day [36,44].

This gain in throughput is particularly valuable for breeding and genetic studies, where phenotyping can become a major bottleneck. Paulsmeyer and colleagues analyzed 398 pigmented maize accessions and found substantial genetic control of anthocyanin content and composition, while Chatham and Juvik combined chromatographic phenotyping with genotyping to identify loci associated with anthocyanin concentration and composition [14,32]. Increasing analytical throughput should therefore enable larger mapping populations and more efficient selection for anthocyanin-rich maize.

The tradeoff is reduced resolution of some structurally similar compounds. While we saw some degree of coelution in the 5-min method, coelution has also been reported in longer maize methods as well, and other groups have similarly used selected ion monitoring to estimate the contribution of coeluting anthocyanins [14,40]. We anticipate that differences seen between methods were likely due to co-elution given that Purple Cónico, a variety with few co-eluting isomers, had nearly identical estimates among methods (Figure 2). We also note that estimates for the analytes surveyed in our method are relative to structurally similar compounds. However, these molecules likely have different ionization efficiencies, and the proportion of anthocyanins in a given variety that can be accounted for with external standards may also heavily influence any differences between the 5- and 25-min methods.

For routine quantification of known major anthocyanins, limited coelution among closely related isomers is an acceptable compromise. This statement is further strengthened by our findings that analytical method generally contributed <1% of the total variance in our population-scale study (Table 3). Profiling a larger population with multiple growing locations would likely shrink the analytical contribution to total variance even further. Given the findings of our validation studies and the lack of authentic standards, the present method is best considered a high-throughput phenotyping platform rather than an exhaustive quantitative method. Future work with more diverse maize populations may allow for the addition of succinylated and condensed flavonoid–anthocyanin molecules to this method.

## 4. Materials and Methods

### 4.1. Chemicals and Reagents

LC–MS grade water, methanol (MeOH), acetonitrile (ACN), and formic acid (FA) were purchased from Fisher Scientific (Pittsburgh, PA, USA). Puerarin (≥98%), cyanidin 3-glucoside (CyG; ≥98%), pelargonidin 3-glucoside (PgdG; ≥97%), and peonidin 3-glucoside (PndG; ≥98%) were purchased from Sigma Aldrich (St. Louis, MO, USA). Strata-X 33 μm polymeric reversed-phase solid-phase extraction (SPE) cartridges (30 mg/3 mL) were purchased from Phenomenex, (Torrance, CA, USA).

### 4.2. Internal Standard and Standard Curve Preparation

To prepare the standard stock solutions for the standard curve, 0.2 ± 0.1 mg of CyG, PgdG, and PndG were weighed, and 2% formic acid (FA) in water was added to each standard to achieve a final concentration of 0.1 mg/mL. To prepare the standard curve, 100 μL of each stock solution, 36 μL of 250 μM puerarin (4% of total volume), and 564 μL of 2% FA in water were added to a 4 mL vial (900 μL total volume). For the six subsequent 4 mL vials used in the serial dilution, 24 μL of 250 μM puerarin (4% total volume) and 576 μL of 2% FA in water were added (600 μL total volume). Serial dilutions were performed by aliquoting 300 μL from the 900 μL stock vial into the first 600 μL dilution vial, pipetting up and down four times to mix, and then repeating the process with subsequent dilution vials up to a 1/729 dilution.

### 4.3. Sample Material

A set of five commercially available heirloom high-anthocyanin maize varieties (Purple Cónico, Pink Xocoyul, Blue Cónico, Yellow Bolita, and White Cacahuazintle) were included as well as TAMZ102, a breeding line from Texas A&M University (CS 7 30D). Sample material was selected based on visible differences in coloration spanning the apparent range of pigmented maize. At least three samples were analyzed from each of these materials for the method validation experiments. Additionally, a population of eleven individual TAMZ102 plants were cultivated in a greenhouse in 3-gallon pots according to Eddy and Hahn [45]. Mature kernels were harvested 30 days after pollination, flash-frozen with liquid nitrogen, and stored at −80 °C before freeze drying [32]. Ceramic mortar and pestles were pre-chilled with liquid nitrogen, and the whole kernels were ground until a fine powder where uniform color was achieved.

### 4.4. Quality Control Material and Pseudo-Standard Preparation

Using the remaining intact kernels, pericarp was removed using hand using razor blades until at least 500 mg of pericarp from each variety was collected in 15 mL snap cap tubes. Pericarp samples were frozen using liquid nitrogen, ground by hand using a glass rod, freeze dried, and stored at −20 C° for subsequent extraction and analysis.

Quality control (QC) composite samples were made by mixing equal weights of either ground freeze-dried whole kernels or pericarp from the corn lines described above. The first set of QC composite samples was used for gradient optimization, while the second set was used for multiple reaction monitoring (MRM) optimization due to the low signal of less common analytes in whole kernels. Both sets of QC composite samples were extracted as described below.

### 4.5. Anthocyanin Extraction

The extraction process used in this study was based on the methodology outlined by Chatham and colleagues with modifications [32]. For each sample, 125 mg ± 2.5 mg was weighed in 15 mL flip-cap tubes, and 2.5 mL of 2% FA in water was added. Samples were bath sonicated for 10 min. Samples were incubated in a water bath at 50 °C for 1 h. Every 15 min during the incubation step, samples were removed from the water bath and manually agitated to dislodge material at the bottom of each tube and improve homogeneity. Samples were centrifuged for 5 min at 3700 RPM and 23 °C (Eppendorf 5920R), and the supernatant for each sample was collected in corresponding 125 mm borosilicate glass tubes. Samples were evaporated in a RapidVap (LabConco; Kansas City, MO, USA) at 30 mbar and 35 °C and kept at −80 °C for future redissolution and analysis. For QC composite samples used for method optimization, solid-phase extraction (SPE) was then performed as described below.

### 4.6. Solid-Phase Extraction (SPE)

Solid-phase extraction (SPE) was performed to concentrate QC composite material and consequentially increase signal for low-abundance analytes during gradient and MRM optimization. Strata-X 33 μm polymeric reversed-phase 30 mg/3 mL cartridges were used as described by Dzakovich and others with minor alterations [46,47]. Anthocyanins were eluted from cartridges with 1 mL MeOH + 1% FA instead of MeOH + 0.1% FA to improve anthocyanin stability. Samples were evaporated to dryness as described above and kept at −80 °C for future redissolution and analysis.

### 4.7. Redissolving and Filtering

Dried QC composite samples were redissolved in 200 μL of 2% FA in water and filtered through 0.22 μm cellulose acetate into 300 μL LC vials. Samples used for quantification were redissolved in 3 mL of 2% FA in water. A 2880 μL sample aliquot was added to 120 μL of 250 μM puerarin internal standard and filtered through 0.2 μm cellulose acetate into 2 mL LC vials.

### 4.8. Columns and Instruments

A ThermoFisher Vanquish Horizon UHPLC coupled to a ThermoFisher TSQ Altis (Thermo Fisher Scientific, Waltham, MA, USA) was used for tandem mass spectrometry. For the Waters (Waters Corporation, Milford, MA, USA) ACQUITY Premier 2.1 × 100 mm BEH C18 1.7 μm column with 5 mm Vanguard FIT guard cartridge (25-min method), the mobile phase consisted of 0.5% formic acid in water (A) and 0.1% FA in ACN (B) at 0.5 mL/min in a gradient from 5% B to 20% B in 12.35 min for anthocyanin elution, a transition to 60% B in 0.1 min, a hold at 60% B for 5 min, a transition back to 5% B in 0.1 min, and re-equilibration at 5% B for 7.45 min. The gradient for the Waters ACQUITY Premier 2.1 × 30 mm BEH C18 1.7 μm column with 5 mm Vanguard FIT guard cartridge (5-min method) used the same mobile phases and flow rate described earlier with a gradient of 5% B to 15% B in 1.2 min, from 15% to 20% B in 1.2 min, a transition to 60% B in 0.06 min, a hold at 60% B for 1.14 min, a transition back to 5% B in 0.06 min, and re-equilibration at 5% B for 1.34 min. The column compartment for both methods was maintained at 35 °C, and 5 μL injection volumes were used for samples and standards.

Analytes were quantified in electrospray ionization positive mode using the following settings: spray voltage, 4600 V (static); sheath gas, 40 (arbitrary units); aux gas, 20 (arbitrary units); sweep gas, 1 (arbitrary unit); ion transfer tube and vaporizer temperatures, 350 °C; Q1 and Q3 resolution, 1.2 peak width at half height; collision gas, 1.5 mTorr; and source fragmentation, 0 V. Specific details on analyte abbreviations, masses, retention times, and other mass spectrometry details can be found in Supplemental Table S1.

### 4.9. Analyte Identification, Integration, and Quantification

Authentic reference standards of cyanidin-3-glucoside, pelargonidin-3-glucoside, and peonidin-3-glucoside were used to confirm the identities of these compounds. For anthocyanins without commercially available reference standards, compound assignments were based on previously characterized maize anthocyanin profiles [37], supported by product ion scans and chromatographic elution order established during method development. Analyte-specific precursor and product ion transitions and retention times for both chromatographic methods are provided in Supplemental Table S1.

Chromatographic peaks were integrated using QuanBrowser (Thermo Fisher Scientific, Waltham, MA, USA) based on the expected retention times and MRM transitions for each analyte. Peaks were assigned to individual anthocyanins when the corresponding chromatographic signals could be sufficiently resolved to permit reproducible integration. Because the 5-min chromatographic method reduced the separation of some structurally related anthocyanins, certain isomers sharing identical precursor and product ion transitions could not be reliably distinguished. Any unresolved signals were integrated collectively and reported as combined anthocyanin groups rather than individual compounds.

Integrated peak areas were normalized to the puerarin internal standard and quantified using calibration curves generated from authentic reference standards corresponding to the respective anthocyanidin backbone. Accordingly, anthocyanins lacking authentic reference standards were quantified as standard equivalents rather than absolute compound-specific concentrations. Information including goodness of fit, linearity range, coefficients of determination, and calibration point deviations for representative standard curves are available in Supplemental Table S2.

### 4.10. Intraday and Interday Variability Experiments

A single operator extracted six technical replicates of Purple Cónico maize over three days using the methodology described above. Each batch of samples was analyzed on a different day by the same operator with an independent standard curve used in each run. Intra- and interday variability was determined by calculating the coefficient of variation for a given analyte within a day or between days, respectively.

### 4.11. Limit of Detection and Quantification

Limits of detection (LOD) and quantification (LOQ) were estimated from three independent calibration curves on three independent days using authentic anthocyanin standards. Calibration curves were generated by plotting analyte/puerarin ratios against moles on column and fitted using weighted linear regression (1/x^2^) [48,49]. LODs and LOQs used cutoffs of 3.3σ/S and 10σ/S, respectively. Here, σ indicates the standard deviation of the y-intercepts from the three calibration curves, while S represents the mean slope of the regressions.

### 4.12. Statistics and Data Visualization

Statistical analyses and data visualization were performed in R [v.4.5.1]. Variance attributable to maize variety, chromatographic method, and their interaction was estimated for individual anthocyanins using linear mixed-effects models implemented with the lme4 package [50]. Models were fitted by restricted maximum likelihood (REML) according to maize variety, chromatographic method, their interaction, and residual. For each analyte, variance components were expressed as percentages of the total estimated variance. Analytes with no observed variation were excluded from variance component estimation.

Principal component analysis (PCA) was conducted using FactoMineR [51] with the package’s default centering and unit variance scaling. Variables containing only zero values were excluded before PCA. Sample scores and variable contributions to the first two principal components were extracted for visualization. PCA scores were grouped by maize variety and chromatographic method for the method comparison experiment.

Interday differences in individual anthocyanins and anthocyanin classes were evaluated using one-way analysis of variance (ANOVA), with analytical day as the independent factor. Model residuals were assessed for normality using the Shapiro–Wilk test, and homogeneity of variance was evaluated using Levene’s test. When comparing days, pairwise differences were evaluated using Tukey’s honestly significant difference procedure, and adjusted *p* values were used to generate compact letter displays at (*α* = 0.05). Zero-valued observations were excluded from these analyte-specific analyses as implemented in the analysis workflow. Analytical precision was additionally summarized as the coefficient of variation (%CV), calculated as 100 × SD/$\bar{x}$. Intraday %CV was calculated independently for six replicate measurements within each of three analytical days and then averaged across days, whereas interday %CV was calculated from all 18 measurements pooled across the three days.

For comparison of total anthocyanin concentrations obtained using the 30 and 100 mm chromatographic methods, maize variety, chromatographic method, and their interaction were evaluated using a two-factor ANOVA. Residual distributions and homoscedasticity were initially assessed using diagnostic residual plots, the Shapiro–Wilk test, and Levene’s test. Because the untransformed data did not satisfy model assumptions, a Box–Cox profile likelihood analysis was used to identify an appropriate transformation. A Box–Cox parameter of λ=1/3 which was near the profile likelihood optimum and within its 95% confidence interval, was selected, and concentrations were transformed as (Y^(1/3)−1)/(1/3). Model assumptions were reassessed following transformation. Estimated marginal means and pairwise contrasts were obtained using the emmeans package [52], including comparisons of chromatographic methods within each variety and comparisons among varieties within each chromatographic method. Tukey adjustment was used for multiple comparisons, and estimated marginal means were back-transformed to the original concentration scale for interpretation. Statistical significance was defined as *p* < 0.05. Figures were generated primarily using ggplot2 and ggpubr and assembled using patchwork [53,54,55].

## 5. Conclusions

Taken together, these results suggest that the greatest strength of our 5-min UHPLC–MS/MS method is to generate phenotypic data rapidly and consistently. Rapid phenotyping methods are critical for applications in plant breeding and genetics, where the analytical bottleneck can restrict population size and therefore statistical power. A rapid assay capable of reproducibly quantifying the predominant maize anthocyanins, even with limited coelution among closely related isomers, provides a complementary tool to slower, high-resolution LC–MS/MS methods used for compound discovery and structural confirmation. Future improvements in extraction recovery, internal standard selection, and matrix effects will further strengthen the method while preserving the throughput that makes it useful for large-scale maize phenotyping.

## Figures and Tables

**Table 1:** Estimates of the limit of detection and limit of quantification for anthocyanins profiled in this method with available commercial standards.

Analyte	LOD (fmol/inj.)	LOQ (fmol/inj.)
Cyanidin-3-glucoside	64.63	195.85
Pelargonidin-3-glucoside	40.20	121.82
Peonidin-3-glucoside	25.64	77.71

**Table 2:** Intraday and interday variability estimates for all quantifiable analytes.

Analyte ^z^	Intraday Variation (%)	Interday Variation (%)
Total cyanidins	4.71	6.76
Total anthocyanins	4.41	6.74
Total peonidins	6.60	8.62
Total pelargonidins	7.02	8.44
PndG 463 m/z	5.95	6.46
PgdG 433 m/z	7.79	8.16
CyM and unknown 535 m/z	5.65	8.51
CyG 449 m/z	4.73	4.86
CyMG 535 m/z	4.61	10.81
PndDMG and unknown 635 m/z	6.55	11.55
CyDMG and unknown 621 m/z	5.18	16.07
PndMG and combined 549 m/z peaks	6.23	12.44
PgdMG 519 m/z	7.53	13.43
PgdDMH and PgdDMG 605 m/z	9.02	13.93
PgdMH isomers #1 and #2 519 m/z	7.01	10.20
AfCydG 883 m/z	ND ^y^	ND
AfPgdG 867 m/z	ND	ND
AfPgdMdG 953 m/z	ND	ND
AfPndG 897 m/z	ND	ND
CaCyMdG 985 m/z	ND	ND
CaCydG 899 m/z	ND	ND
CaPgdG 883 m/z	ND	ND
CaPgdMdG 969 m/z	ND	ND
CaPndMdG 999 m/z	ND	ND
CatPndG 913 m/z	ND	ND
PgdDMH 605 m/z	NQ ^x^	NQ

^z^ Analyte abbreviations are defined in Supplemental Table S1. ^y^ ND = not detected (below limit of detection). ^x^ NQ = not quantified (below limit of quantification).

**Table 3:** Outputs from random-effects modeling separating the contributions toward total variance due to plant genetic background, analytical method used, the interaction between these terms, and residual error.

Analyte ^z^	Genetic Background (%)	Analytical Method (%)	Interaction (%)	Residual (%)
Total anthocyanins	94.5	0.5	4.9	0.1
Total cyanidins	95.4	0.3	4.2	0.1
Total peonidins	93.2	0.6	6.0	0.2
Total pelargonidins	89.4	2.0	8.1	0.5
PndG 463 m/z	99.8	0.0	0.0	0.2
PgdG 433 m/z	99.7	0.0	0.0	0.3
CyM 535 m/z	98.0	0.2	1.7	0.1
CyG 449 m/z	97.4	0.3	2.2	0.1
CyMG 535 m/z	96.2	0.7	3.0	0.1
PndDMG 635 m/z	92.5	0.2	7.1	0.2
CyDMG 621 m/z	90.0	0.8	9.1	0.1
PndMG 549 m/z	89.5	1.6	8.7	0.2
PgdMG 519 m/z	88.1	3.2	8.2	0.5
PgdDMH and PgdDMG 605 m/z ^y^	87.2	1.8	10.7	0.3
PgdMH 519 m/z	70.9	3.5	12.5	13.1

^z^ Analyte abbreviations are defined in Supplemental Table S1. ^y^ Analytes co-eluted in the 5-min method and their aggregate sum was used for analysis.

## Data Availability

The original contributions presented in this study are included in the article/Supplementary Materials. Further inquiries can be directed to the corresponding author.

## Supplementary Materials

The following supporting information can be downloaded at: https://www.mdpi.com/article/10.3390/molecules31213920/s1, Figure S1: Diversity and range of individual quantified anthocyanin concentrations. Boxplots illustrate concentrations expressed as mg/100 g; Figure S2: Chromatograms of individual anthocyanin analytes in a representative sample. Chromatograms from the 5-min (orange) and 25-min (blue) methods are overlayed highlighting differences in chromatographic resolution between methods. Table S1: Summary of multiple reaction monitoring information and analyte identities, Table S2: Summary information for representative standard curves (5-min method) generated for validation experiments, Table S3: Raw data used for intraday and interday variability experiments, Table S4: Intraday and interday variation data for the 25-min (100 mm) method, Table S5: Raw data used for random effects modeling comparing the 5- and 25-min methods, Table S6: Anthocyanin data from nine TAMZ102 individuals profiled using the 5-min and 25-min methods.

## Abbreviations

The following abbreviations are used in this manuscript:

ACN	Acetonitrile
BEH	Bridged ethylene hybrid
CyG	Cyandin-3-glucoside
DAD	Diode array detector
DW	Dry weight
FA	Formic acid
HPLC	High-performance liquid chromatography
LC–MS/MS	Liquid chromatography–tandem mass spectrometry
LOD	Limit of detection
LOQ	Limit of quantification
MRM	Multiple reaction monitoring
NMR	Nuclear magnetic resonance
PCA	Principal component analysis
PDA	Photodiode array detector
PgdG	Pelargonidin-3-glucoside
PnDG	Peonidin-3-glucoside
REML	Restricted maximum likelihood
SPE	Solid-phase extraction
UHPLC	Ultra-high-performance liquid chromatography
